# Barriers and enablers to providing healthy food and beverages in New Zealand secondary school canteens

**DOI:** 10.1093/heapro/daaf011

**Published:** 2025-02-26

**Authors:** Olivia Rose Coady, Sara Elizabeth Styles, Claire Smith

**Affiliations:** Te Tari Kai Tōtika Takata, Ōtākou Whakaihu Waka, Ōtepoti 9054, Aotearoa; Department of Human Nutrition, University of Otago, Dunedin 9054, New Zealand; Te Tari Kai Tōtika Takata, Ōtākou Whakaihu Waka, Ōtepoti 9054, Aotearoa; Department of Human Nutrition, University of Otago, Dunedin 9054, New Zealand; Te Tari Kai Tōtika Takata, Ōtākou Whakaihu Waka, Ōtepoti 9054, Aotearoa; Department of Human Nutrition, University of Otago, Dunedin 9054, New Zealand

**Keywords:** food, health policy, health-promoting environments, qualitative methods, school

## Abstract

School environments have the potential to promote healthy dietary behaviours among adolescents. In New Zealand, there is no regulation regarding the healthiness of foods and beverages available to purchase at school canteens. This qualitative study explored the barriers and enablers to providing healthy food and beverages in secondary school canteens. An electronic screening questionnaire was distributed to all secondary schools to identify schools with a canteen. Semi-structured interviews among participants representing purposively selected schools explored the experiences of providing healthier foods and beverages in the canteen. Among schools (*n* = 333) invited to participate in the survey, 78 schools (response rate 23.4%) responded, with 37 schools reporting a canteen onsite. Of these, 24 schools were purposively invited to participate. Ten interviews were completed with participants representing six schools and one interview with an external food service provider. Through reflexive thematic analysis, four key themes were identified: (i) an action-oriented over-arching school policy based on healthy eating principles facilitates healthier provisions, (ii) fully supported initiatives across the school environment facilitate healthier provisions, (iii) champions facilitate healthier school canteens, and (iv) healthy canteens are not prioritized within the school’s broader needs. School canteens are more likely to follow healthy eating principles when food and beverage policies are clear and comprehensive, adequate resources are available to implement and sustain healthier options, champions are involved, and the canteen is part of a whole-school approach that benefits the overall school food environment.

**Contribution to Health Promotion:**

- This study used rigorous qualitative research methods, informed by the socio-ecological model, to explore the barriers and enablers that influence the provision of healthy food and beverages in secondary school canteens.
- Our findings highlight the importance of a champion to advocate for healthier canteen provisions and adequate financial resources and staff time to ensure the availability of healthy food and beverages in secondary school canteens.
- There is a clear need for sustainable funding to support creating and maintaining healthier school environments.
- Nutrition policies must be clear and their implementation monitored.
- Mandatory features of policies will foster better adherence to healthy eating principles.

## INTRODUCTION

The Lancet Commission on Adolescent Health and Well-being states that adolescents are an important but neglected age group (Patton et al. 2016). Healthy dietary practices during adolescence can have an immediate impact on academic performance, health and well-being, and long-term impacts including prevention of non-communicable diseases later in life (World Health Organization 2020). Globally, adolescents’ consumption of fruits and vegetables, sodium, fat, sugar-sweetened beverages, and water is misaligned with dietary guidelines and recommended nutrient intakes (Diethelm et al. 2012, Beal et al. 2019).

Schools are ideal settings to promote healthy dietary behaviours (World Health Organization 2020). Adolescents consume 9%–35% of their daily energy during school hours (Regan et al. 2008, Lachat et al. 2012, Tugault-Lafleur et al. 2017, Valizadeh and Ng 2020). Canteens (tuck-shops) are an important aspect of a whole-school system approach to healthy eating in schools (Drummond and Sheppard 2011). However, less healthy foods and beverages are often available for students to purchase (Woods et al. 2014, Azeredo et al. 2016, Nortje et al. 2017, D’Souza et al. 2022), potentially undermining healthy food environment initiatives (França et al. 2022). While there currently is no one-size-fits-all approach to creating healthy food environments in schools, policies aimed at school food services, including canteens, have been identified as a promizing strategy to improve students’ dietary behaviours (Micha et al. 2018, Vandevijvere et al. 2019). However, many schools struggle to adhere to healthy food and beverage policies due to financial, physical, and social factors (Ronto et al. 2020).

### New Zealand food environment in secondary schools

In New Zealand, ~292 000 adolescents attend secondary education from Year 9 to Year 13 (Ministry of Education., 2023a, Education Counts 2024). Most (83%) secondary schools sell food and/or beverages through an internally operated service or an external provider (D’Souza et al. 2022). While many New Zealand school canteens sell whole grain sandwiches, wraps, fruit, and water, they also sell less healthy items such as pastries, sausages, American hot dogs, ice cream, juice, and other sweet snacks (D’Souza et al. 2022).

In 2019, the New Zealand government launched the Healthy Active Learning initiative as part of its Child and Youth Well-Being Strategy (New Zealand Government 2019). Concurrently, the New Zealand Ministry of Health published the *Healthy Food and Drink Guidance—Schools* (Guidance) to support schools in developing a voluntary policy to promote and provide healthy foods and drinks across the whole school (Ministry of Health 2020). The Guidance is based on three healthy eating principles: (i) offer a variety of healthy foods from the four food groups; (ii) prepare food with minimal saturated fat, salt, and added sugar that should be mostly whole or less processed; and (iii) offer only water and unflavoured milk as drink options. The Guidance classifies foods into three categories: green, amber, and red. Items in the green category are nutrient-rich and lower in saturated fat, salt, and added sugars. The amber category includes processed foods that provide some nutritional value. Foods in the red category are lower in nutritional value and higher in saturated fat, salt, or added sugars. Schools are encouraged to ensure that over 75% of available foods fall within the green category (Ministry of Health 2020). Additionally, the healthiness of packaged foods is evaluated by the Health Star Rating—a front-of-pack label that assesses nutritional quality. Packaged foods with a Health Star Rating of 3.5 or higher are considered healthier options compared with similar products (Ministry of Health 2020). However, only a minority of secondary schools adhere to this guideline (Chote et al. 2022). Reasons for low adherence to the Guidance include resistance from students and families and loss of profits (Chote et al. 2022).

While a healthy food and beverage policy is voluntary for schools, the Guidance is mandatory for schools serving free lunches through the nationwide programme, Ka Ora, Ka Ako (Ministry of Education., 2022). The programme was launched in 2019 to reduce food insecurity as part of the Healthy Active Learning initiative. As of May 2024, 1013 schools and kura (schools where teaching is based on indigenous New Zealanders’ culture and values) were participating in the programme (Ministry of Education 2024). Lunches are audited regardless of whether the school or an external operator provides them. The effects of the free lunch programme are emerging (Chote et al. 2022). Currently, it is not known if the availability of free lunches impacts the food and beverages sold in secondary school canteens.

This study explored the perceptions of key relevant people (an alternative term for ‘stakeholders’; Khuri et al. 2024) regarding the barriers and enablers to providing foods and beverages in the ‘green’ category in secondary school canteens. The results are expected to provide insight into tailoring secondary schools’ initiatives to promote healthy dietary behaviour.

## MATERIALS AND METHODS

### Study design

The research took place within a paradigmatic framework of interpretivism. In this qualitative study, interviews were conducted to understand the complex and interrelated secondary school food environment factors shaping the shared experiences of promoting and providing healthier canteen menu items. The student researcher (O.C.) was also a physical education teacher in a school ineligible for this study, and their subjectivity was viewed as a resource at all stages of the research. This research approach can enable more realistic recommendations to inform healthier food and beverage policies in secondary schools (Neve et al. 2021). Ethical approval for this research was granted by the University of Otago Human Ethics Committee (reference no D21/389).

### Participants and recruitment

In January 2022, New Zealand secondary schools were invited via email to participate in an electronic survey that aimed to identify schools with a canteen, determine the presence of a canteen-specific food and beverage policy as well as common foods and beverages sold in the canteen, and sought schools’ permission to be contacted to participate in a qualitative study about the canteen. The publicly available Ministry of Education directory (https://www.educationcounts.govt.nz/directories/list-of-nz-schools) was used to obtain schools’ contact details and characteristics [e.g. students’ gender, authority (i.e. ownership of the school by the state, private, or state-integrated), area type (considering population size), and presence of boarding facilities]. The directory also provided the socioeconomic position of the school’s student community with decile 1 schools being the 10% of schools with the highest proportion of students from low socioeconomic communities and decile 10 schools being the 10% of schools with the lowest proportion of students from low socioeconomic communities (Ministry of Education., 2023b). Schools with a missing email address or only enrolling students in Years 7 to 11 (11–15 years of age) were excluded. Thus, 333/351 (95%) of secondary schools were invited to participate in the screening survey.

The survey was administered using Research Electronic Data Capture (REDCap) hosted at the University of Otago. REDCap is a secure, web-based software platform created to support secure data capture (Harris et al. 2009, 2019). Responders were instructed to answer the questions in relation to normal circumstances to avoid canteen provision changes resulting from the COVID-19 pandemic. Up to 2 follow-up emails, 1 and 2 months after the initial email were sent to non-responder schools (Dillman et al. 2014). All schools that responded to the survey were entered into a draw for a NZ$200 gift voucher.

From the schools that responded and had a canteen (*n* = 37), 24 schools were purposively invited to participate in the qualitative study. To maximize variability in participating schools, schools were selected based on key characteristics such as gender composition, decile rating, participation in the Ka Ora, Ka Ako program, rurality, and availability of both ‘green’ and ‘red’ items. Of these schools, 15 did not respond, 2 withdrew for COVID-19 pandemic-related reasons, and 1 declined. Additionally, one external provider participated to address gaps in insights among schools that did not internally operate their canteens. Pragmatic decisions determined the final sample size (i.e. time available to the student researcher, the narrow focus on secondary school canteens, the desired diversity of the sample, and the richness of interview data) (Braun and Clarke 2021). Participants provided written informed consent and consent from their school’s principal (where relevant) before the interview.

### Data collection

The student researcher conducted 1:1 semi-structured interviews (*n* = 10) via Zoom between February and May 2022. All participants were aware that the student researcher was also a teacher trained in New Zealand and working overseas. The first interview was supervised by an experienced qualitative researcher (S.S.). A further interview (*n* = 1) was conducted via email at the participant’s request, with all questions and subsequent requests for clarification emailed to the participant.

The student researcher developed the interview guide (see Supplementary File) drawing from literature on healthy food provision in Australian and New Zealand canteens (Lawlis et al. 2016, Ronto et al. 2020, D’Souza et al. 2022, McPherson et al. 2022) and their experiences as a teacher, including reviewing her school’s food and beverage policy. The interview guide was refined iteratively after pretesting with two teachers, three senior leadership team members, and one canteen staff member, which were not used in the analysis. Minor revisions were made to the interview structure, relevance, and clarity of questions, and procedures for conducting interviews using Zoom. Further topics were added to the guide after the third, fourth, and sixth interviews as new insights emerged, including the influence of Ka Ora, Ka Ako on canteen provisions and the operation of the canteen.

The interviewer kept field notes as a record of observations and the context of each interview as well as key points and recurring patterns across the interviews. Interviews lasted up to 1 hour were recorded and transcribed by Otter.ai (https://otter.ai), checked for accuracy, and returned to participants for review. No participant revised their transcript. Participants received an NZ$50 supermarket voucher as a token of appreciation.

### Data analysis

Descriptive statistics were calculated to summarize participants’ and schools’ characteristics. Reflexive thematic analysis (Braun and Clarke 2022), following a rigorous six-phase process, facilitated a deeper exploration of the research aim. The student researcher conducted the analysis beginning with data familiarization (phase 1). This involved checking transcripts for accuracy, taking notes on new patterns and meanings, and rereading transcripts throughout the process to ensure all data were considered. The next step involved generating codes (phase 2) from sections of meaningful text. Initially, coding using NVivo 12 qualitative data analysis software was deductive. It was informed by ideas from previous research on school canteens and the student researcher’s experience as a teacher (e.g. ‘champions’ being individuals that facilitate healthy canteen provisions). Coding was also semantic, reflecting the explicit content of the data (e.g. ‘healthy food is expensive’). Later, transcripts were re-read, and coding became more inductive, reflecting the content of the data. This included insights such as the influence of the free lunch programme. It also became latent, reflecting assumptions underlying the overt content of the data [e.g. ‘healthy eating is (not) a priority’]. To generate themes (phase 3), a mind map was created to capture shared meaning between codes. These were grouped by levels of the socio-ecological framework (McLeroy et al. 1988). This framework suggests that behaviour is determined by interrelated factors. These include intrapersonal factors, interpersonal processes, organizational factors, community factors, and policy factors, which can influence each other. Researchers collaboratively revised the themes (phase 4) by reviewing all participant quotes from relevant codes, ensuring that themes were supported by quotes across all participants. These themes were defined (phase 5) to capture the theme’s essence and ensure each theme had boundaries. As part of the report production (phase 6), data extracts (quotes) were selected to illustrate each theme.

## RESULTS

Eleven interviews were conducted among 10 participants representing 6 schools, and 1 interview with an external provider responsible for developing canteen menus. Participants’ roles in schools included employed canteen staff (*n* = 3), catering manager (*n* = 1), principal/assistant principal (*n* = 3), business manager/executive officer (*n* = 2), and school nurse (*n* = 1). All schools were Government (state) owned. Most schools were coeducational (*n* = 4; all-girls, *n* = 1; all-boys, *n* = 1), located in main urban areas (*n* = 4), did not provide boarding facilities (*n* = 4), were part of the Ka Ora, Ka Ako free lunch programme (*n* = 4), and operated their canteen internally (*n* = 4). The schools’ socioeconomic statuses ranged from schools with the highest proportion of students from low socioeconomic communities (‘deciles 1–3’, *n* = 3) to schools with the lowest proportion of students from low socioeconomic communities (‘deciles 7–10’, *n* = 1). Three schools had a policy explicitly informed by the Guidance and used to make decisions about the types of foods and beverages sold in the canteen.

Four key themes (Table 1) were identified as prominent barriers and enablers to healthy food and beverages being sold in secondary school canteens: (i) an action-oriented over-arching school policy based on healthy eating principles facilitates healthier provisions, (ii) fully supported initiatives across the school environment facilitate healthier provisions, (iii) champions facilitate healthy canteens, and (iv) healthy canteens are not prioritized within the school’s broader needs. The impact of Ka Ora, Ka Ako on the wider school environment, including the canteen, was a salient factor among schools participating in this programme.

**Table 1.** Themes and subthemes influencing the healthiness of New Zealand secondary school canteen provisions

Themes	Impact
An action-oriented, over-arching, and mandatory school policy based on healthy eating principles facilitates healthier food and beverages	Policies provide a middle ground between key relevant people, particularly when they are prescriptive about how to implement healthy eating principles and enforceable.
Fully supported initiatives across the school environment facilitate healthier provisions	Mandates, funding, practical support, monitoring, and incentives enable schools to provide healthier foods and beverages in canteens and create a healthier school environment.
Champions facilitate healthy canteens *Subtheme* A collaborative approach can overcome barriers to healthy canteens.	Champions (in any relevant role) value nutritious foods and beverages, have a positive attitude toward healthier canteen provisions, believe in the benefits of providing healthier provisions, and are capable of contributing to a healthier canteen—they are a catalyst for healthier canteen options.
Healthy canteens are not prioritized within the school’s broader needs	Other factors related to students’ well-being and education can be more important to principals/senior leadership teams to address within the school’s limited resources, leading to healthier canteen provisions being a lower priority.

### Theme 1: An action-oriented, over-arching, and mandatory school policy based on healthy eating principles facilitates healthier provisions

Participants described the role a policy has or could have in influencing school decisions. Comprehensive policies offering clear guidance on healthy food and beverage selection streamlined decision-making among key relevant people. As noted by an external food provider, a policy provides a foundation for decisions.

The school has a firm nutrition policy, so they will be like, ‘There can only be water.’ They’ll pick through what’s on our menu and say, ‘Not that. Not that. Not that.’

Participants from two schools distributing free lunches through the Ka Ora, Ka Ako programme described how implementing the Guidance influenced what was sold in the canteen. The prescriptive requirements were an effective bargaining tool when working with suppliers, leading to the reformulation of some recipes. The following quote describes a conversation about making a recipe healthier:

‘Your (supplier) current meatballs are categorised in the ‘red’ category of the traffic light system, which we’re not allowed to put any ‘red’ category items in our lunch boxes, and our lunch boxes need to be 75% ‘green’ category.’ Gives a real clear framework around that kind of thing. So, they reformulated their meatballs to be ‘amber’. (External Provider)

In general, participants viewed mandatory policies as more effective than voluntary ones in enforcing healthy eating principles, with greater adherence observed. Two participants recalled the effectiveness of earlier national mandatory legislation on school canteens, which resulted in large improvements to the healthiness of foods sold in the canteen; however, the legislation was later withdrawn. One participant recalled,

We went to Heart Tick (reference for a healthier version of a product) pies and got rid of a whole lot of cream on things… Then we were just free reign again. We could do what we wanted to sell to the kids. (Canteen Employee)

Interestingly, the Ka Ora, Ka Ako requirements did not always extend to a school’s canteen, even though their food and beverage policy included it. Instead, one participant described meeting with the school principal to discuss what was considered ‘healthy’ for the school canteen. This school had several items on their canteen menu that were less healthy, such as hot chips, fruit juices, hashbrowns, and pies. Selling foods that were not allowed in Ka Ora, Ka Ako lunches meant schools could cater to students’ tastes through the canteen.

### Theme 2: Fully supported initiatives across the school environment facilitate healthier provisions

Resource availability significantly influenced canteen offerings, with limited resources posing challenges in providing both compliant lunches (for Ka Ora, Ka Ako) alongside healthy canteen options. Financial viability and uncertainty regarding Ka Ora, Ka Ako funding due to changes in the New Zealand Government deterred investment in equipment for the school canteen.

I think it’s (Ka Ora, Ka Ako) going for at least another year and a half, I believe, or maybe at the end of two, then …it could be something that could be cut… So, we’re not in a position to invest, really. (Deputy Head Teacher)

Some participants described staff being reallocated from the school canteen to make lunches compliant with Ka Ora, Ka Ako. Prioritizing staffing for the free lunch programme contributed to an increase in the availability of ‘amber’ and ‘red’ foods in the canteen. One participant explained the impact of staff constraints:

…we have slipped a little, and we’re sort of in a bit of a dilemma now because now we’ve got the healthy kai (food, for Ka Ora, Ka Ako) and the canteen. The cafe lady just hasn’t got the time. (Assistant Principal)

Fit-for-purpose cooking and food preparation facilities did enable some schools to prepare healthier food. However, one participant described their experience with inadequate food preparation and storage facilities. While the school had a refrigerator, there was not enough space to store ingredients for healthier foods, beverages, and food ready to be sold. Reduced operational costs for internally operated canteens enabled schools to provide or continue to provide healthier food compared with externally operated canteens, which face additional costs such as rent or insurance. Participants agreed that financial barriers, particularly ensuring the canteen is profitable, impacted their ability to provide healthy food. For example, a school nurse commented, ‘It’s easier to serve greasy cheap food, and moneywise, it makes the business (canteen) more viable, apparently’.

### Theme 3: Champions facilitate healthy canteens

This theme highlights the values, attitudes, and capabilities of ‘champions’. Champions held many roles in the school, including Board of Trustee members, Senior Leadership Team, teachers, and canteen staff. Champions played a vital role in driving initiatives to improve canteen environments, often overcoming resource constraints through collaboration and advocacy for healthier options. One champion described their longer term vision of the food and beverage policy they helped introduce:

If I was to leave…we’ve put the borders in place… You know, we don’t add other stuff (unhealthy food) back in. We’ve been successful at something, don’t undo it. (Canteen Manager)

Champions had previous experiences, education, and skills to create a healthy food environment in the canteen. However, there was no consistent pattern of a particular workshop, study, or previous employment. For example, one participant completed a university paper on nutrition promotion, whereas another participant acquired experience writing policies and creating menus through working at a playcentre, being a nanny and working at the school.

Participants were unaware of any professional development opportunities within schools related to improving the healthiness of foods and beverages sold in the canteen. Therefore, professional development always took place outside the school environment.

Student taste preferences helped and hindered a champion’s ability to ensure a healthy food environment in the canteen, but more often, it was a commonly reported hindrance. Participants shared a view that champions were happy to provide healthy food if students purchased it, but this was not always the case.

I made salads and healthy sandwiches, sushi, hot pasta and sausage rolls, which I made from scratch with apples and vegetables in, but unfortunately the kids really want crap food at their canteen. (Canteen Employee)

One champion stopped trying to make a positive change due to lacking support and collaboration from key relevant people. The participant explained,

I’m willing to get involved (in helping the canteen be healthier), but you know… it just gets too hard. You kind of get pushed out, and you think, ‘Well, I’ve got too many other things to do’. (Assistant Principal)

This champion had tried many different avenues to improve the school canteen. They explained,

We brought it (canteen food) up for a Year 12 health project once where they can pick a health topic and try to change it in the school, and we just got shut down really quickly, and then I got told, can we just ‘leave this alone’. (Acting Assistant Principal)

#### Subtheme: A collaborative approach can overcome barriers to healthy canteens

Participants highlighted that collaboration within and across school groups or key relevant people could facilitate a healthier canteen. The benefits of collaboration included increasing buy-in from students, enabling the sharing of knowledge and skills, receiving support from senior leadership, and managing resource constraints (e.g. money). One participant explained,

We (canteen staff) work alongside the nurses…. So we looked at (the menu) with the traffic light system… You just do simple things like a wrap is ‘green’ but making it even better by swapping it out for your wholegrain and instead of your white bread. (Canteen Manager)

This school also worked with the students who were part of the ‘Hauora (Well Being) Council’. This council reviewed ‘amber’ and ‘red’ food items and suggested ‘green’ items to sell in the canteen. They also used posters to encourage healthy eating choices.

One school used the expertise of the school’s departments to provide healthy food. For example, a teacher from food and technology helped the canteen with tips on how the canteen staff could make healthier foods more affordable.

It was common for parents to be involved in the school food provision and influence decision-making. Involvement included parents serving as members of the Board of Trustees, parents working in the canteen and supporting Ka Ora, Ka Ako. To illustrate the influence at the Board of Trustee level, one participant shared the impact of parent values:

I’m not a parent, but people on the Board are, and the principal is a parent, and you know things like the discussion was around ‘Would we be happy with our child, you know, eating that (unhealthy food) every now and then, every once in a while?’ Those were the discussions around what we decided to have in the canteen. (Catering Manager)

### Theme 4: Healthy canteens are not prioritized within the school’s broader needs

This theme describes the reality faced by schools as they juggle multiple priorities. Most participants believed that healthy eating was essential, but other factors related to students’ well-being and education were more important to address with schools’ limited resources. One participant explains:

Higher rates of truancy and absenteeism… we’re dealing with higher rates of bullying, and I would say the negative effects of technology. We’re dealing with higher mental health issues for young people, and I think that when you think about all of those things, they, for a lot of schools, they are what takes precedence over, ‘Okay, let’s make sure our kids are eating healthy food’. (Principal)

The types of food and beverages offered in the school canteens were influenced by what nearby food outlets were selling. Four schools decided to sell less healthy foods to compete with nearby food outlets, keep students on school grounds, and maintain sales within canteens. One participant explained,

The school has become fluid about the food as the students started to go off-site and get food (from a nearby fast food outlet) and they would get Uber Eats sometimes to deliver cheap pizzas. So, we do now sell pies and try our best to stick with wholesome other foods. (Assistant Principal)

Not all schools chose to provide less healthy food as a strategy to reduce truancy. One participant explains how they instead chose to compete financially:

You can go and buy a Big Mac at McDonald’s for, I don’t know, say $12. But you can buy sushi here (at the school) for $8, or you can buy macaroni and cheese for $8. (Executive Officer)

However, when a school considers healthy eating a priority, they will do what is needed to overcome the barriers. As explained by one participant,

The Board is very clear that at a minimum, we just need to break even so that we can make sure that our students have opportunity to buy food. (Principal)

Considering why this school took such measures, the principal stated their values and priorities for the school were ‘quality (food)… something that’s good and healthy, that’s good sustenance in it’, which was followed by ‘the bottom line’.

## DISCUSSION

Adolescents spend a considerable amount of time at school, thus making schools an important health promotion setting. The complexity of implementing and maintaining effective healthy food and beverage policies in schools has been recognized in international research (McIsaac et al. 2019). Informed by the socio-ecological model, this qualitative study considered multiple levels of influence on providing healthy food and beverages in secondary school canteens. The research was timely because some schools simultaneously participated in the newly implemented free lunch programme and operated their canteen. The findings underscore the importance of comprehensive, mandatory school food, and beverage policies that align with healthy eating principles. These policies should be supported by adequate resources and champions who advocate and assist schools in implementation along with a collaborative, whole-school approach. This research also highlighted how competing priorities may overshadow the healthiness of foods and beverages sold in the canteen.

Nutrition policies are a way schools can support students’ health and well-being (Chriqui et al. 2014). International research suggests unambiguous, enforceable policies that are clearly communicated to key relevant people are enablers in implementing and complying with school food and beverage policies (Ronto et al. 2020). However, the effectiveness of healthy food and beverage policies can be undermined by students’ access elsewhere in school (e.g. vending machines or fundraisers) or in the immediate vicinity (e.g. convenience stores) (Pillay et al. 2023). Furthermore, the voluntary nature of policies and a lack of monitoring and evaluation contribute to low compliance with school nutrition policies (Woods et al. 2014, Ronto et al. 2020). Monitoring school canteens is considered a factor that can contribute to long-term adherence to healthy food and beverage policies and is therefore recommended (Johnston et al. 2022). Auditing has been implemented within the free lunch programme(Mejia Toro and Swinburn 2024); thus, extending monitoring to school canteens may be feasible.

Our findings highlight the importance of adequate financial resources and staff time in ensuring the availability of healthy food and beverages in secondary school canteens. Schools operating a canteen and Ka Ora, Ka Ako reported stretched resources hampering a school-wide healthy food environment. The costs associated with implementing a healthy food and beverage policy, including food expenses, equipment procurement, and staff training, pose barriers to providing healthy food and beverages in schools (Pettigrew et al. 2014, Ronto et al. 2020). International research has shown that financial pressures often lead schools to compromise on nutrition particularly when government funding is insufficient (Story et al. 2009). Thus, governments clearly need to allocate sustainable funding to support the creation of healthier school environments.

The important role of champions advocating for healthier canteen options was evident. Champions were individuals in any relevant role who felt compelled to uphold their value of nutritious food despite challenges. Others’ attitudes toward healthy food and beverages undermined champions’ efforts and impacted policy implementation. Previous research has found negative attitudes commonly hinder progress in providing healthier options (Ronto et al. 2020). Similarly, teachers’ and canteen staff’s knowledge of healthy eating principles may need to be taught or refreshed through professional development (Bryan et al. 2019, Ronto et al. 2020). The values upheld by champions prioritize health over profit and are similar to key relevant peoples’ values in Australian research (McPherson et al. 2022). In their research, McPherson et al. (2022) suggest that such values contribute to schools’ increased commitment to following healthy food and beverage guidance. Schools that use the canteen for promoting health messages often integrate nutrition education into school curricula, fostering a culture of valuing health (McPherson et al. 2022). However, the pressure for schools to generate profit from their canteens can lead to selling less healthy but popular items, posing a widespread challenge (Pillay et al. 2023). Proposals to price healthier options competitively aim to address this issue (O’Halloran et al. 2020). Nonetheless, the need for school canteens to prioritize profits should be critically examined in light of the power imbalance between school food services and the food industry. Hawkins and Rundle (2024) have argued that the modern food system, dominated by multinational food companies, promotes highly processed and unhealthy foods, making it challenging for school food to compete effectively.

Consistent with the literature (Ronto et al. 2020), a common barrier to healthier canteen provisions was other competing priorities. In this study, truancy was a priority and previous research identified schools experiencing pressure to prioritize improving academic performance (Ronto et al. 2020). Furthermore, at the time of this study, schools were still coping with the unprecedented challenges of teaching during the COVID-19 pandemic (Buckley Flack et al. 2020, Sharp et al. 2020). A potential solution is integrating the canteen into a whole-school approach to overall health and well-being (United Nations Educational Scientific and Cultural Organization 2023). This is exemplified in initiatives such as ‘Fruit in Schools’, which offers a free piece of fruit each day to students attending primary schools in the lowest socioeconomic neighbourhoods (Boyd et al. 2007). Participating schools integrate healthy eating principles into the curriculum and empower students to ‘learn by doing’ as they take action on a healthier lifestyle (e.g. students create canteen menu items within resource constraints), which could impact dietary choices outside the food environment (Pillay et al. 2023).

## STRENGTHS AND LIMITATIONS

This study used rigorous qualitative research methods to gain insights into the socio-ecological influences on providing healthy food in secondary school canteens from a range of key relevant peoples’ perspectives. This study complements a recent review of the Ka Ora, Ka Ako free lunch programme’s impacts across five social levels from the individual students to the broader food systems (Garton et al. 2023). In order to better provide nutritious food and beverages to students through both the free lunch programme and the school canteen, it is clear that some challenges (e.g. administrative burden due to more staff, purchasing food, management of the kitchen) and opportunities (e.g. new government food procurement models, partnering with local producers) should be considered. Maximum variability in the characteristics of participating interviewees and the schools they represent may help the transferability of the findings to secondary schools across New Zealand but may limit transferability to international contexts. There is a possibility that interviewees and the schools they represented shared commonalities (e.g. views, experiences, characteristics) that differed from those who did not participate. The themes should be interpreted with consideration given to other limitations. One participant completed their interview via email; while this facilitated their study participation, their responses may not have been as in-depth had the interview been conducted face-to-face. Some participants may not have fully disclosed their canteen operation out of fear of undermining the school principal or board. Finally, the study did not include parents or students, which are important groups for future school health and nutrition policy research, as they could influence schools’ priorities.

## CONCLUSION

This research has identified key factors that influence the provision of healthy food in New Zealand secondary school canteens. An action-oriented, over-arching healthy food and beverage policy can enable the provision of healthy foods and beverages. Resource constraints and competing priorities can hinder the provision of healthy food. Dedicated ‘champions’ play an important role in advocating and implementing policies. Strong policies that are clear and comprehensive, adequate resources, and professional development for school staff are essential to improving and creating healthy canteens.

## Supplementary Material

daaf011_suppl_Supplementary_Material

## Data Availability

The data underlying this article cannot be shared publicly for the privacy of individuals who participated in the study.
