# Supplementary material for: Barriers and enablers to providing healthy food and beverages in New Zealand secondary school canteens
Source: Health Promot Int. 2025 Feb 26;40(1):daaf011. doi: 10.1093/heapro/daaf011 (PMC11878539; doi:10.1093/heapro/daaf011)
Supplement: daaf011_suppl_Supplementary_Material [file daaf011_suppl_supplementary_material.docx]

**The barriers and enablers to providing healthy food in New Zealand secondary school canteens: Supplementary material**

1. Interview guide

**Semi-structured interview guide**

| **Topic** | **Question** |
| --- | --- |
| Participant | |
| Role in running or making decisions about canteen | Tell me about your role at school and your role within the canteen.  *Board of Trustees member*  What is your role on the board?  Tell me more about the Board’s role in the school food provision and canteen.  *Senior Leadership Team member*  How did you come to oversee the canteen/food provision? |
| School canteen | |
| Day-to-day operation | Tell me about the day-to-day running of the school canteen.  *Prompts*  Is your school’s canteen operated internally by the school or by an external provider?  How is the canteen funded?  Is your school’s canteen for-profit?  Is your school’s canteen run by volunteers or paid staff?  Tell me about the facilities used to prepare, cook, and serve the food.  Have any recent changes to your school’s canteen been made?  *Canteen staff*  Tell me what your job involves in a typical day.  Tell me about the types of food you prepare in a day.  How important is it to make a profit?  *Board of Trustees member and Senior Leadership Team member*  Tell me about the funding the school receives towards the canteen?  What is the canteen profit used for?  Does the Board/Senior Leadership Team consider the canteen facilities in the strategic planning?  *Senior Leadership Team member*  Does the school provide funding towards the operation of the canteen?  *Externally operated canteen - Board of Trustees member and Senior Leadership Team member*  Why did your school hire that particular food supplier?  Were there particular criteria that your school had to meet when the food supplier was first hired?  Describe what support you receive from the food suppliers to provide healthy food. |
| School canteen menu | What factors are important when deciding what the canteen sells?  What, if any, guidelines are used when deciding what the canteen sells?  *Prompts*  What are the barriers to providing “healthy” (‘green’) foods?  What are the barriers to banning “unhealthy” (red) foods?  Have you heard of the Ministry of Education’s Healthy Eating Guidelines?  Does your school use the Ministry of Education’s Healthy Eating Guidelines?  What would help your school canteen implement the Ministry of Education’s Healthy Eating Guidelines?  *External provider/operator*  What factors are important to your company?  Does the school encourage you to use the Ministry of Education’s Healthy Eating Guidelines?  Does your company encourage you to use the Ministry of Education’s Healthy Eating Guidelines? |
| School food and nutrition policy | Does the school have a food and nutrition policy?  Prompts  How helpful or unhelpful is the policy?  *OR*  How helpful or unhelpful would a policy be? |
| Professional development/Skills and knowledge | *Canteen staff*  Tell me about the skills required to work in the canteen.  Tell me about the training you received regarding nutrition education.  Tell me about the (nutrition-specific) resources or support your school/company provides. |
